# Correlation of Genetic Variants and the Incidence, Prevalence and Mortality Rates of Acute Lymphoblastic Leukemia

**DOI:** 10.3390/jpm12030370

**Published:** 2022-02-28

**Authors:** Marianne Rodrigues Fernandes, Lui Wallacy Morikawa Souza Vinagre, Juliana Carla Gomes Rodrigues, Alayde Vieira Wanderley, Sweny Marinho Fernandes, Laura Patrícia Albarello Gellen, Angélica Leite de Alcântara, Beatriz Brilhante de Sousa, Rommel Mario Rodríguez Burbano, Paulo Pimentel de Assumpção, Sidney Emanuel Batista dos Santos, Ney Pereira Carneiro dos Santos

**Affiliations:** 1 Research Center of Oncology, Federal University of Pará Belém, Belém 66073-000, PA, Brazil; luivinagre@gmail.com (L.W.M.S.V.); julianacgrodrigues@gmail.com (J.C.G.R.); alaydevieira@yahoo.com.br (A.V.W.); swenymf@gmail.com (S.M.F.); laura.patricia.agellen@hotmail.com (L.P.A.G.); angelica.alcantara99@gmail.com (A.L.d.A.); beatriz.sousa@icb.ufpa.br (B.B.d.S.); assumpcaopp@gmail.com (P.P.d.A.); sidneysantosufpa@gmail.com (S.E.B.d.S.); npcsantos@yahoo.com.br (N.P.C.d.S.); 2 Otávio Lobo Children’s Cancer Hospital-HOIOL-Belém, Belém 66063-005, PA, Brazil; rommelburbano@gmail.com

**Keywords:** acute lymphoblastic leukemia, susceptibility, epidemiological data

## Abstract

Acute lymphoblastic leukemia (ALL) is the most common cancer during childhood, representing about 30–35% of cases. Its etiology is complex and not fully understood. ALL is influenced by genetic variants, and their frequencies (Fq) vary in different ethnic groups, which consequently could influence the epidemiology of this cancer worldwide. The aim of this study was to investigate the correlation between the genetic variants and their impacts on incidence (IC), mortality (MT), and prevalence (PV) rates of ALL in different world populations. Methods: Sixty variants were selected from the literature with Genome Wide Association studies (GWAS). Information regarding allele Fq was selected from the 1000 Genomes platform. Epidemiological data were taken from the Global Burden of disease visualisations (GBD) Compare website. Statistical analyses were calculated in RStudio v.3.5.1 software. Results: Four variants demonstrated significant results in correlations with epidemiological data for ALL. The *PAX5* gene variant (rs2297105) had an indirect relationship with PV and IC of ALL, showing that an increased Fq of this variant is related to low rates of both. An increased Fq of rs915172 in *EPB4IL2* gene was also correlated with a lower IC of ALL. The rs1048943 of the *CYP1A1* gene and the rs3088440 polymorphism of the *CDKN2A* gene were shown to have a direct proportional relationship with MT rate, showing that an increased Fq of these variants correlates with a worse prognosis worldwide. Conclusion: Our study points out four important variants for understanding the IC, PV, and MT rates for ALL. The ascertainment of these data may help to choose molecular markers to investigate the susceptibility and prognosis of ALL.

## 1. Introduction

Acute lymphoblastic leukemia (ALL) is the most common malignant cancer in childhood, representing about 30–35% of cases. The leukemogenesis process can occur in both B and T lymphoid lineages, which can invade the bone marrow, peripheral blood, and tissues [1].

Incidence and lethality rates of childhood ALL vary among countries [2]. In 2017, the global incidence of ALL was 1.04/100,000, while the global mortality was 0.72/100,000. In this analysis, the American continent had the highest incidence and mortality rates (1.45/100,000 and 1.03/100,000, respectively) [3].

ALL’s etiology is complex and not fully understood, however, it is known to be associated with both endogenous and exogenous factors. Confirmed risk factors include congenital cytogenetic disorders and exposure to various environmental factors, such as certain viral infections. However, these factors can explain only about 10% of all cases, while the remaining continue with their origins unknown [4,5].

Recent Genome Wide Association Studies (GWAS) have identified different genetic variants associated with higher risk for ALL, including single nucleotide polymorphisms (SNPs) in important genes, suggesting this type of leukemia could relate to a polygenic susceptibility [6,7,8,9]. However, there is no consensus in these studies in worldwide populations.

Another factor associated with the incidence of ALL is the ethnic characteristics of different populations [7]. This disparity observed between populations occurs due to the presence of genetic polymorphisms and intrinsic environmental factors to which these groups are exposed [10]. Some studies have already highlighted Amerindian ancestry and mixed populations as risk factors for developing of toxicity in ALL [9,11,12,13]. Therefore, our study aimed to investigate the correlation between genetic polymorphisms involved in biological pathways for ALL and their impacts on incidence, mortality, and prevalence rates in different world populations.

## 2. Materials and Methods

### 2.1. Variant Selection and Genetic Data Collection

Polymorphisms were selected from the literature by searching the PubMed platform with the following descriptors: “Acute lymphoblastic leukemia”, “GWAS”, “risk”, “susceptibility”, “population”, which led to 46 articles. Inclusion criteria were: Variants found in GWAS and meta-analysis studies, variants associated with protection, susceptibility, or risk to ALL. We obtained 60 polymorphisms described for the development of acute lymphoblastic leukemia (Supplementary Table S1). Among these, 56 were excluded because they did not significantly correlate with acute lymphoblastic leukemia.

### 2.2. Frequency Information on Analyzed Variants

Variants’ frequency information was obtained from the 1000 Genomes Project platform (Available online: https://www.internationalgenome.org/ (Accessed on 5 October 2021), available in Phase 3 Release of the 1000 Genomes Database

Specific frequencies were collected for each ethnic group described in the platform: African (AFR), American Mixed (AMR), Asian (EAS), European (EUR), and South Asian (SAS).

### 2.3. Epidemiological Data Collection

Data on incidence (IC), prevalence (PV), and mortality (MT) rates of ALL in global populations were obtained from the Global Burden of disease visualisations (GBD) Compare website, created by the Institute for Health Metrics and Evaluation (http://vizhub.healthdata.org/gbd-compare, accessed on 28 January 2022). We collected the epidemiological rates from all countries analyzed in 1000 Genomes database (Supplementary Table S2).

### 2.4. Statistical Analysis and Plots

Correlation analysis between frequencies of genetic variants and epidemiological rates was calculated by Pearson’s correlation coefficient. All analyses were performed in RStudio v.3.5.1 software.

## 3. Results

A total of 60 polymorphisms were analyzed regarding their association with IC, MT and/or PV rates of ALL. Among these, four (rs2297105, rs915172, rs1048943, and rs3088440) showed significance in the correlation analyses. Information about these variants is available in Table 1.

**Figure 1:**
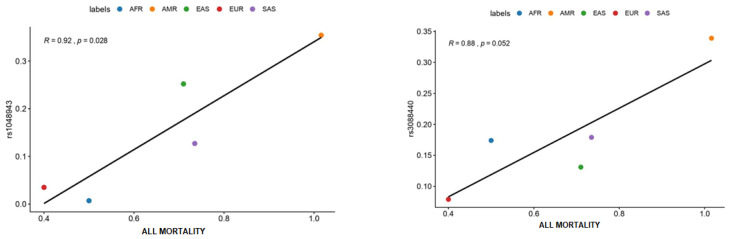
Mortality rates of ALL. **Abbreviations:** African (AFR), American Mixed (AMR), Asian (EAS), European (EUR), and South Asian (SAS), Acute lymphoblastic leukemia (ALL).

Figure 1 shows that two variants–rs1048943 of the *CYP1A1* gene and rs3088440 of the *CDKN2A* gene–were positively correlated with mortality rates. Both images show homogeneity in our findings, since the higher the frequency of the variant allele in these polymorphisms, the higher were mortality rates (showed as per million of individuals). Based on this correlation, AMR individuals have higher frequencies of the variant alleles, and, consequently, have higher rates of ALL mortality. On the other hand, European populations have the lowest frequency of these variants, also having the lowest mortality rates.

**Figure 2:**
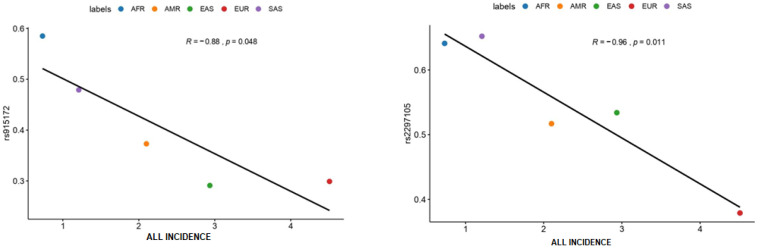
ALL Incidence Rates. **Abbreviations:** African (AFR), American Mixed (AMR), Asian (EAS), European (EUR), and South Asian (SAS), Acute lymphoblastic leukemia (ALL).

Regarding incidence (Figure 2), we found that both rs2297105 of the *PAX5* gene and rs915172 of *EPB41L2* gene are negatively correlated with ALL incidences. AFR and SAS have the highest frequencies of both these variants, also having the lowest rates of ALL incidences, while EUR has the lowest frequency and the highest incidence. This finding may indicate a possible protective factor regarding these variants.

**Figure 3:**
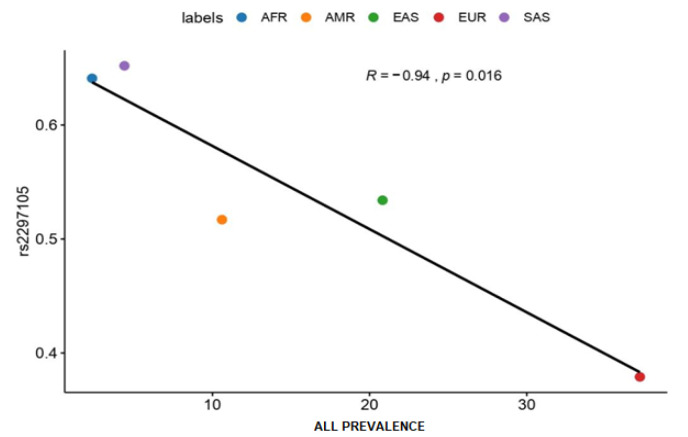
ALL Prevalence Rates. **Abbreviations:** African (AFR), American Mixed (AMR), Asian (EAS), European (EUR), and South Asian (SAS), Acute lymphoblastic leukemia (ALL).

Regarding prevalence rates (Figure 3), only one polymorphism showed a statistically significant inverse correlation (rs2297105 of the *PAX5* gene). According to these results, the EUR and EAS populations have lower frequencies of these variant alleles, which grant them higher ALL prevalence rates.

## 4. Discussion

A large fluctuation in ALL’s epidemiological rates is observed worldwide [1]. One of the risk factors that may contribute to this variability is the inherent genetic factors of each ethnicity [7,10,11]. Several genomic studies with a large number of patients show that genetic variants may act as risk factors for ALL in populations with different patterns of ethnic composition [13,14].

This study gathered the IC, PV, and MT rates found in countries compounding the 1000 Genomes database and correlated these data with the allele frequencies of genetic variants, which have already been associated with susceptibility for ALL GWAS studies. Our results show a strong correlation of four genetic variants (rs2297105, rs915172, rs1048943, and rs3088440) in four genes (*PAX5, EPB41L2, CYP1A1,* and *CDKN2A*) with PV, IC, and MT rates.

The *PAX5* gene encodes the paired box protein PAX5, a member of the family of transcription factors essential for B-cell differentiation since it activates specific genes and represses those that compromise hematopoietic lineages [15]. Thus, PAX5 is one of the proteins with a high frequency of genetic alterations in B-cell ALL. Such alterations are the results of deletions, translocations, or mutations. About one-third of cancers are consequences of *PAX5* gene modifications, and the complete loss of this gene is related to the highest aggressiveness of B-cell ALL [16,17].

Our results show that a higher frequency of the polymorphism rs2297105 in the *PAX5* gene is associated with a lower IC and a lower PV of ALL. The rs2297105 variant in the *PAX5* gene showed fluctuations between frequencies of the polymorphism in the different populations, as we found high frequency in the African population (0.641) and low frequency in Europeans (0.379). However, the frequency of rs2297195 was inversely associated with the prevalence and incidence of ALL, as the European population showed higher prevalence and incidence of ALL when compared to the African population. This polymorphism has been associated as a susceptibility locus for ischemic or hemorrhagic stroke [14] and glucocorticoid resistance in ALL [18].

The EPB41L2 is a member of the 4.1 proteins, encoded by the *EPB41* (erythrocyte membrane protein band 4.1-like 2) genes, which are components of the cortical cytoskeleton underlying the cell membrane [19]. The EPB41 family form nodes in the cell cortex, connecting further components of the cortical cytoskeleton–like spectrins, actin, and transmembrane adhesion proteins, receptors, and transporters [20]. Polymorphisms in the *EPB41L2* gene has been associated with childhood acute lymphoblastic in genome-wide association study in Korea [6].

In our study, the rs915172 variant of the *EPB41L2* gene showed an association with ALL incidences, as the lower the frequency of this variant, the higher the incidence for ALL. In our study, the rs915172 variant of the *EPB41L2* gene showed an association with ALL incidences, as the lower the frequency of this variant, the higher the incidence for ALL. We found the highest frequency in the African population (0.585), which was associated with lower incidence of ALL, while the opposite happened with the European population, which showed a lower frequency of the SNP (0.299) and a higher incidence of ALL. This result may suggest the polymorphism as a protective factor for susceptibility to ALL. This is the first study to correlate this specific polymorphism with a pathological condition, for which further clinical studies are required to clarify their relationship.

The *CYP1A1* gene is a member of the CYP family that participates in the metabolism and elimination of exogenous chemical compounds. Genetic alterations can modify enzyme activity and compromise the metabolism of mutagenic substances. Polymorphisms in these enzymes positively influence susceptibility to ALL in children [21]. Most studies investigating the role of this gene in leukemia susceptibility are done in Oriental populations [22]. A meta-analysis has associated the presence of this polymorphism with an increased risk for developing ALL and Acute Myeloid Leukemia in Caucasian individuals [23].

Our findings also include a positive correlation between the rs1048943 polymorphism of the *CYP1A1* gene with ALL mortality rates, given that, the higher the frequency of this variant, the higher the ALL mortality rates. The highest frequency was found in the American population (0.354), and the lowest in European (0.035).

The cyclin-dependent kinase 2A inhibitor (*CDKN2A*) encodes two proteins important for cell cycle regulation: p14ARF, which binds to *p53* and inhibits *MDM2*, and p16INK4a, which is a cyclin-dependent kinase inhibitor. Both act as tumor suppressors [24]. Mutations in the *CDKN2A* gene have been observed in several types of cancers, such as melanoma-like, pancreatic, intestinal, prostate, and gastric lymphoma [25]. Deletions in the *CDKN2A/B* gene have been correlated with a worse prognosis of ALL in patients of all ages, and a missense variant associated with the unique risk of ALL in European and Hispanic children has been identified, conferring a threefold increased risk for ALL [26,27].

In our study, the rs3088440 variant in the *CDKN2A/B* gene was positively associated with MT rate, so the higher the frequency of the variant allele, the higher the numbers of ALL’s incidence and mortality. The highest frequency was found in the American population (0.354), and the lowest in European (0.127).

In the present study, it was not possible to stratify the populations investigated according to the socioeconomic level of the patients and/or their levels of access to medical resources. This limitation made it possible to include unique ethnic groups with relevant data for the analyses.

## 5. Conclusions

Large genome-wide studies are important for identifying variants associated with susceptibility to complex diseases. However, the results found may differ among populations worldwide due to fluctuation in allele frequencies of these variants among different ethnic groups. Our study aimed to correlate data on genetic variants associated with ALL with the epidemiology rates of this disease.

Our results point to four important variants (rs2297105, rs915172, rs1048943, and rs3088440) for understanding the incidence, prevalence, and mortality rates associated with this cancer. The rs1048943 and rs3088440 variants were positively associated with ALL mortality, and both variants were more frequent in the American population and less frequent in the European population.

On the other hand, the rs9151172, rs2297105 variants were inversely related to the incidence rates of ALL, with higher frequency in the African population and lower frequency in the European population. The ascertainment of these data may help to choose molecular markers to investigate the susceptibility and prognosis of ALL.

## Figures and Tables

**Table 1:** Information about the significant variants investigated.

Position	Gene	Variant ID	Location	Impact Predicted by SNPeff	Variable
9: 37020625	*PAX5*	rs2297105	Intronic	Modifier	Incidence and prevalence
6: 130863630	*EPB41L2*	rs915172	Splice site region	Low	Incidence
15: 74720644	*CYP1A1*	rs1048943	Non-synonymous coding	Moderate	Mortality
9: 21968160	*CDKN2A*	rs3088440	3′-UTR	Modifier	Mortality

**Abbreviations:** Genetic variant annotation and functional effect prediction toolbox (SNPeff).

## Data Availability

All relevant data will be shared as Supporting Information files if the manuscript is accepted for publication.

## Supplementary Materials

The following supporting information can be downloaded at: https://www.mdpi.com/article/10.3390/jpm12030370/s1, Table S1: Studied Genes and References; Table S2: Epidemiological Data on Global Population.
