# Supplementary material for: Correlation of Genetic Variants and the Incidence, Prevalence and Mortality Rates of Acute Lymphoblastic Leukemia
Source: J Pers Med. 2022 Feb 28;12(3):370. doi: 10.3390/jpm12030370 (PMC8954641; doi:10.3390/jpm12030370)
Supplement: Supplementary file 1 [file jpm-12-00370-s001.zip › jpm-1520760-supplementary.pdf]

Correlation of Genetic Variants and The Incidence, Prevalence, and Mortality Rates of Acute Lymphoblastic Leukemia

Supplementary Materials:

Table S1: Studied Genes and References

GENES	REFERENCES
ARID5B	Gutierrez-Camino A, Martin-Guerrero I, García-Orad A. Genetic susceptibility in childhood acute lymphoblastic leukemia. Med Oncol. 2017. Sep 13;34(10):179. doi: 10.1007/s12032-017-1038-7. PMID: 28905228.<br><br>Hsu LI, Briggs F, Shao X, Metayer C, Wiemels JL, Chokkalingam AP, Barcellos LF. Pathway Analysis of Genome-wide Association Study in Childhood Leukemia among Hispanics. Cancer Epidemiol Biomarkers Prev. 2016 May;25(5):815-22. doi: 10.1158/1055-9965.EPI-15-0528.<br><br>Papaemmanuil E, Hosking FJ, Vijayakrishnan J, Price A, Olver B, Sheridan E, Kinsey SE, Lightfoot T, Roman E, Irving JA, Allan JM, Tomlinson IP, Taylor M, Greaves M, Houlston RS. Loci on 7p12.2, 10q21.2 and 14q11.2 are associated with risk of childhood acute lymphoblastic leukemia. Nat Genet. 2009 Sep;41(9):1006-10. doi: 10.1038/ng.430.
CDKN2A, CDKN2B, CDKN1B	Gutierrez-Camino A, Martin-Guerrero I, García-Orad A. Genetic susceptibility in childhood acute lymphoblastic leukemia. Med Oncol. 2017 Sep 13;34(10):179. doi: 10.1007/s12032-017-1038-7. Sherborne AL, Hosking FJ, Prasad RB, ET AL. Variation in CDKN2A at 9p21.3 influences childhood acute lymphoblastic leukemia risk. Nat Genet. 2010 Jun;42(6):492-4. doi: 10.1038/ng.585.<br><br>Hsu LI, Briggs F, Shao X, Metayer C, Wiemels JL, Chokkalingam AP, Barcellos LF. Pathway Analysis of Genome-wide Association Study in Childhood Leukemia among Hispanics. Cancer Epidemiol Biomarkers Prev. 2016 May;25(5):815-22. doi: 10.1158/1055-9965.EPI-15-0528.
CEBPE	Zou ZQ, Yue LJ, Ren YF. [Association between CYP1A1*2A polymorphism and susceptibility to childhood acute lymphoblastic leukemia: a Meta analysis]. Zhongguo Dang Dai Er Ke Za Zhi. 2015 Oct;17(10):1112-8.<br><br>Gutierrez-Camino A, Martin-Guerrero I, García-Orad A. Genetic susceptibility in childhood acute lymphoblastic leukemia. Med Oncol. 2017 Sep 13;34(10):179. doi: 10.1007/s12032-017-1038-7.

CYP1A1	Zou ZQ, Yue LJ, Ren YF. [Association between CYP1A1*2A polymorphism and susceptibility to childhood acute lymphoblastic leukemia: a Meta analysis]. Zhongguo Dang Dai Er Ke Za Zhi. 2015 Oct;17(10):1112-8.<br><br>Brisson GD, Alves LR, Pombo-de-Oliveira MS. Genetic susceptibility in childhood acute leukaemias: a systematic review. Ecancermedicalscience. 2015 May 14;9:539. doi: 10.3332/ecancer.2015.539.<br><br>Vijayakrishnan J, Houlston RS. Candidate gene association studies and risk of childhood acute lymphoblastic leukemia: a systematic review and meta-analysis. Haematologica. 2010 Aug;95(8):1405-14. doi: 10.3324/haematol.2010.022095.
CYP2D6	Silveira Vda S, Canalle R, Scrideli CA, Queiroz RG, Tone LG. Role of the CYP2D6, EPHX1, MPO, and NQO1 genes in the susceptibility to acute lymphoblastic leukemia in Brazilian children. Environ Mol Mutagen. 2010 Jan;51(1):48-56. doi: 10.1002/em.20510.
CYP2E1	Brisson GD, Alves LR, Pombo-de-Oliveira MS. Genetic susceptibility in childhood acute leukaemias: a systematic review. Ecancermedicalscience. 2015 May 14;9:539. doi: 10.3332/ecancer.2015.539.<br><br>Vijayakrishnan J, Houlston RS. Candidate gene association studies and risk of childhood acute lymphoblastic leukemia: a systematic review and meta-analysis. Haematologica. 2010 Aug;95(8):1405-14. doi: 10.3324/haematol.2010.022095.
CYP3A5	a LM, Liu HC, Ruan LH, Feng YM. CYP3A5 * 3 genetic polymorphism is associated with childhood acute lymphoblastic leukemia risk: A meta-analysis. Biomed J. 2015 Sep-Oct;38(5):428-32. doi: 10.4103/2319-4170.151029.
ELK3	Gutierrez-Camino A, Martin-Guerrero I, García-Orad A. Genetic susceptibility in childhood acute lymphoblastic leukemia. Med Oncol. 2017 Sep 13;34(10):179. doi: 10.1007/s12032-017-1038-7.
EPB41L2	Han S, Lee KM, Park SK, Lee JE, Ahn HS, Shin HY, Kang HJ, Koo HH, Seo JJ, Choi JE, Ahn YO, Kang D. Genome-wide association study of childhood acute lymphoblastic leukemia in Korea. Leuk Res. 2010 Oct;34(10):1271-4. doi: 10.1016/j.leukres.2010.02.001.

ERG	Semmes EC, Vijayakrishnan J, Zhang C, Hurst JH, Houlston RS, Walsh KM. Leveraging Genome and Phenome-Wide Association Studies to Investigate Genetic Risk of Acute Lymphoblastic Leukemia. Cancer Epidemiol Biomarkers Prev. 2020 Aug;29(8):1606-1614. doi: 10.1158/1055-9965.EPI-20-0113.
FPGS	Piwkham D, Siriboonpiputtana T, Beuten J, Pakakasama S, Gelfond JA, Paisooksantivatana K, Tomlinson GE, Rerkamnuaychoke B. Mutation Screening and Association Study of the Folylpolyglutamate Synthetase (FPGS) Gene with Susceptibility to Childhood Acute Lymphoblastic Leukemia. Asian Pac J Cancer Prev. 2015;16(11):4727-32. doi: 10.7314/apjcp.2015.16.11.4727.
GATA3	Hsu LI, Briggs F, Shao X, Metayer C, Wiemels JL, Chokkalingam AP, Barcellos LF. Pathway Analysis of Genome-wide Association Study in Childhood Leukemia among Hispanics. Cancer Epidemiol Biomarkers Prev. 2016 May;25(5):815-22. doi: 10.1158/1055-9965.EPI-15-0528.
GSTM1	Brisson GD, Alves LR, Pombo-de-Oliveira MS. Genetic susceptibility in childhood acute leukaemias: a systematic review. Ecancermedicalscience. 2015 May 14;9:539. doi:10.3332/ecancer.2015.539.<br><br>hen HC, Hu WX, Liu QX, Li WK, Chen FZ, Rao ZZ, Liu XF, Luo YP, Cao YF. Genetic polymorphisms of metabolic enzymes CYP1A1, CYP2D6, GSTM1 and GSTT1 and leukemia susceptibility. Eur J Cancer Prev. 2008 Jun;17(3):251-8. doi: 10.1097/CEJ.0b013e3282b72093.<br><br>Zhao T, Ma F, Yin F. Role of polymorphisms of GSTM1, GSTT1 and GSTP1 Ile105Val in childhood acute lymphoblastic leukemia risk: an updated meta-analysis. Minerva Pediatr. 2018 Apr;70(2):185-196. doi: 10.23736/S0026-4946.17.04657-6.
GSTP1	Al-Eitan LN, Rababa'h DM, Alkhatib RQ, Khasawneh RH, ALjarrah OA. GSTM1 and GSTP1 Genetic Polymorphisms and Their Associations with Acute Lymphoblastic Leukemia Susceptibility in a Jordanian Population.
GSTT1	Zhao T, Ma F, Yin F. Role of polymorphisms of GSTM1, GSTT1 and GSTP1 Ile105Val in childhood acute lymphoblastic leukemia risk: an updated meta-analysis. Minerva Pediatr. 2018 Apr;70(2):185-196. doi: 10.23736/S0026-4946.17.04657-6.

HAO1	Han S, Lee KM, Park SK, Lee JE, Ahn HS, Shin HY, Kang HJ, Koo HH, Seo JJ, Choi JE, Ahn YO, Kang D. Genome-wide association study of childhood acute lymphoblastic leukemia in Korea. Leuk Res. 2010 Oct;34(10):1271-4. doi: 10.1016/j.leukres.2010.02.001.
IKZF1	Hsu LI, Briggs F, Shao X, Metayer C, Wiemels JL, Chokkalingam AP, Barcellos LF. Pathway Analysis of Genome-wide Association Study in Childhood Leukemia among Hispanics. Cancer Epidemiol Biomarkers Prev. 2016 May;25(5):815-22. doi: 10.1158/1055-9965.EPI-15-0528.<br><br>Papaemmanuil E, Hosking FJ, Vijayakrishnan J, Price A, Olver B, Sheridan E, Kinsey SE, Lightfoot T, Roman E, Irving JA, Allan JM, Tomlinson IP, Taylor M, Greaves M, Houlston RS. Loci on 7p12.2, 10q21.2 and 14q11.2 are associated with risk of childhood acute lymphoblastic leukemia. Nat Genet. 2009 Sep;41(9):1006-10. doi: 10.1038/ng.430.
IRF1	Semmes EC, Vijayakrishnan J, Zhang C, Hurst JH, Houlston RS, Walsh KM. Leveraging Genome and Phenome-Wide Association Studies to Investigate Genetic Risk of Acute Lymphoblastic Leukemia. Cancer Epidemiol Biomarkers Prev. 2020 Aug;29(8):1606-1614. doi: 10.1158/1055-9965.EPI-20-0113.
LHPP	Gutierrez-Camino A, Martin-Guerrero I, García-Orad A. Genetic susceptibility in childhood acute lymphoblastic leukemia. Med Oncol. 2017 Sep 13;34(10):179. doi: 10.1007/s12032-017-1038-7.
MAN2A1	Han S, Lee KM, Park SK, Lee JE, Ahn HS, Shin HY, Kang HJ, Koo HH, Seo JJ, Choi JE, Ahn YO, Kang D. Genome-wide association study of childhood acute lymphoblastic leukemia in Korea. Leuk Res. 2010 Oct;34(10):1271-4. doi: 10.1016/j.leukres.2010.02.001.
MDR1(ABCB1)	Brisson GD, Alves LR, Pombo-de-Oliveira MS. Genetic susceptibility in childhood acute leukaemias: a systematic review. Ecancermedicalscience. 2015 May 14;9:539. doi: 10.3332/ecancer.2015.539.
MPO	Silveira Vda S, Canalle R, Scrideli CA, Queiroz RG, Tone LG. Role of the CYP2D6, EPHX1, MPO, and NQO1 genes in the susceptibility to acute lymphoblastic leukemia in Brazilian children. Environ Mol Mutagen. 2010 Jan;51(1):48-56. doi: 10.1002/em.20510.

MTHFR	Yan J, Yin M, Dreyer ZE, Scheurer ME, Kamdar K, Wei Q, Okcu MF. A meta-analysis of MTHFR C677T and A1298C polymorphisms and risk of acute lymphoblastic leukemia in children. Pediatr Blood Cancer. 2012 Apr;58(4):513-8. doi: 10.1002/pbc.23137.<br><br>Gómez-Gómez Y, Organista-Nava J, Villanueva-Flores F, Estrada-Brito JS, Rivera-Ramírez AB, Saavedra-Herrera MV, Jiménez-López MA, Illades-Aguiar B, Leyva-Vázquez MA. Association Between the 5,10-MTHFR 677C>T and RFC1 80G>A Polymorphisms and Acute Lymphoblastic Leukemia. Arch Med Res. 2019 May;50(4):175-180. doi: 10.1016/j.arcmed.2019.07.010.
NAT2	Brisson GD, Alves LR, Pombo-de-Oliveira MS. Genetic susceptibility in childhood acute leukaemias: a systematic review. Ecancermedicalscience. 2015 May 14;9:539. doi: 10.3332/ecancer.2015.539.
NQO1	Brisson GD, Alves LR, Pombo-de-Oliveira MS. Genetic susceptibility in childhood acute leukaemias: a systematic review. Ecancermedicalscience. 2015 May 14;9:539. doi: 10.3332/ecancer.2015.539.<br><br>Silveira Vda S, Canalle R, Scrideli CA, Queiroz RG, Tone LG. Role of the CYP2D6, EPHX1, MPO, and NQO1 genes in the susceptibility to acute lymphoblastic leukemia in Brazilian children. Environ Mol Mutagen. 2010 Jan;51(1):48-56. doi: 10.1002/em.20510.<br><br>Vijayakrishnan J, Houlston RS. Candidate gene association studies and risk of childhood acute lymphoblastic leukemia: a systematic review and meta-analysis. Haematologica. 2010 Aug;95(8):1405-14. doi: 10.3324/haematol.2010.022095.
PAX5	Khalid A, Aslam S, Ahmed M, Hasnain S, Aslam A. Risk assessment of FLT3 and PAX5 variants in B-acute lymphoblastic leukemia: a case-control study in a Pakistani cohort. PeerJ. 2019 Sep 10;7:e7195. doi: 10.7717/peerj.7195. PMID: 31565544; PMCID: PMC6743442.
PIP4K2	Hsu LI, Briggs F, Shao X, Metayer C, Wiemels JL, Chokkalingam AP, Barcellos LF. Pathway Analysis of Genome-wide Association Study in Childhood Leukemia among Hispanics. Cancer Epidemiol Biomarkers Prev. 2016 May;25(5):815-22. doi: 10.1158/1055-9965.EPI-15-0528.<br><br>Gutierrez-Camino A, Martin-Guerrero I, García-Orad A. Genetic susceptibility in childhood acute lymphoblastic leukemia. Med Oncol.

	2017 Sep 13;34(10):179. doi: 10.1007/s12032-017-1038-7.
RFC1	Vijayakrishnan J, Houlston RS. Candidate gene association studies and risk of childhood acute lymphoblastic leukemia: a systematic review and meta-analysis. Haematologica. 2010 Aug;95(8):1405-14. doi: 10.3324/haematol.2010.022095.<br><br>Gómez-Gómez Y, Organista-Nava J, Villanueva-Flores F, Estrada-Brito JS, Rivera-Ramírez AB, Saavedra-Herrera MV, Jiménez-López MA, Illades-Aguiar B, Leyva-Vázquez MA. Association Between the 5,10-MTHFR 677C>T and RFC1 80G>A Polymorphisms and Acute Lymphoblastic Leukemia. Arch Med Res. 2019 May;50(4):175-180. doi: 10.1016/j.arcmed.2019.07.010.
SHMT1	Vijayakrishnan J, Houlston RS. Candidate gene association studies and risk of childhood acute lymphoblastic leukemia: a systematic review and meta-analysis. Haematologica. 2010 Aug;95(8):1405-14. doi: 10.3324/haematol.2010.022095.<br><br>Bahari G, Hashemi M, Naderi M, Sadeghi-Bojd S, Taheri M. Association of SHMT1 gene polymorphisms with the risk of childhood acute lymphoblastic leukemia in a sample of Iranian population. Cell Mol Biol (Noisy-le-grand). 2016
XRCC1	Brisson GD, Alves LR, Pombo-de-Oliveira MS. Genetic susceptibility in childhood acute leukaemias: a systematic review. Ecancermedicalscience. 2015 May 14;9:539. doi: 10.3332/ecancer.2015.539.

Table S2: Epidemiological Data on Global Population.

	INCIDENCE	PREVALENCE	MORTALITY
**AMR**			
Califórnia (USA)	1.33	9.21	0.38
Puerto Rico	1.45	9.73	0.44
Colombia	3.39	15.36	1.83
Peru	2.23	8.15	1.42
**EAS**			
China	3.7	26.62	0.91
Vietnam	1.45	5.33	0.9
Japan	3.69	30.55	0.32
**SAS**			

Texas (USA)	0.94	5.51	0.37
Pakistan	1.8	5.13	1.2
Bangladesh	0.89	2.56	0.64
**EUR**			
Utah (USA)	0.96	6.17	0.33
Italy	6.8	56.59	0.54
Finland	4.58	37.92	0.4
Scotland	4.03	33.34	0.35
Spain	6.5	54.19	0.48
England	4.19	34.98	0.3
**AFR**			
Nigeria	0.56	1.6	0.37
Kenya	0.62	1.83	0.45
Gambia	0.45	1.32	0.33
Sierra Leone	1.18	3.4	0.85
Barbados	0.86	3.56	0.5

Data acquires from Global Burden of Disease. **Abbreviations:** African (AFR), American Mixed (AMR), Asian (EAS), European (EUR), and South Asian (SAS).
